# Time Course of the Neural Activity Related to Behavioral Decision-Making as Revealed by Event-Related Potentials

**DOI:** 10.3389/fnbeh.2019.00191

**Published:** 2019-09-03

**Authors:** José M. Martínez-Selva, Miguel A. Muñoz, Juan P. Sánchez-Navarro, César Walteros, Pedro Montoya

**Affiliations:** ^1^School of Psychology, University of Murcia and Murcia Institute for Biomedical Research (IMIB-Arrixaca), Murcia, Spain; ^2^Brain, Mind and Behavior Research Center, University of Granada (CIMCYC-UGR), Granada, Spain; ^3^Research Institute on Health Sciences (IUNICS), University of Balearic Islands (UIB), Palma, Spain

**Keywords:** decision making, evoked potentials, feedback learning, P200 evoked potentials, P300 component

## Abstract

**Objective**: To study the time course of the electrocortical activity evoked by gains and losses in the Iowa Gambling Task (IGT), the brain sources of this electrical activity, and its association with behavioral parameters of task performance in order to achieve a better knowledge of decision-making processes.

**Method**: Event-related potentials (ERPs) were obtained from a 64-channel EEG in 25 participants when performing the IGT. Brain source localization analyses of the ERP components were also assessed.

**Results**: ERP amplitudes were sensitive to gains and losses. An early fronto-central negativity was elicited when feedback was provided for both gains and losses, and correlated with the number of gains at FCz and with the number of both gains and losses at Cz. The P200 component had larger amplitudes to losses and correlated positively with the number of losses. Feedback related negativity (FRN) was higher at frontal, temporal and occipital electrodes in trials with monetary losses. In addition, trials with monetary losses elicited larger P300 magnitudes than trials with monetary gains at all electrode localizations.

**Conclusions**: All ERP components (except P300) were related to participants’ performance in the IGT. Amplitudes of P200 and P300 were associated with the conscious recognition of the error during the decision-making. Performance data and source analysis underline the importance of the medial prefrontal cortex when processing feedback about monetary losses in the IGT.

## Introduction

A current approach for studying reward processing in decision-making involves the use of gambling tasks in which participants’ decisions may result in some form of monetary gain and loss. These tasks have been used in both behavioral and neuroimaging studies of healthy participants, as well as neurological and psychiatric patients. In addition, the recording of event-related potentials (ERPs) during the processing of choice outcomes may provide insight into the time course of neural responses to reward and punishment. This is especially important in those tasks used in clinical settings such as the Iowa Gambling Task (IGT; Bechara et al., 1994).

The IGT is a useful tool to study real-life decision-making under uncertainty and has proved to be useful in the detection of decision-making impairments in several neurological and psychiatric conditions (Bechara et al., 1994, 2001; Bolla et al., 2003; Tchanturia et al., 2007; Buelow and Suhr, 2009; Walteros et al., 2011). The IGT consists of 100 trials in which participants are asked to select a card from one of the four decks at a time. The four decks have different monetary gains and losses that vary in quantity and probability, such that two of them offer high immediate gains, but larger losses in the long term (disadvantageous decks), while the remaining two decks offer immediate gains lower, but also lower long-term losses (advantageous decks). Participants must adjust their options by learning from the feedback they receive immediately after each election, which are the advantageous and disadvantageous decks. Therefore, this is a complex decision-making task in which decisions are made under great ambiguity and unpredictability, particularly (but not exclusively) during the first 40 trials due to lack of knowledge about the probabilities of winning and loss associated with each deck.

Several neuroimaging and brain injury studies indicate that IGT performance is related to the integrity and activity of different areas of the brain, located mainly in the frontal lobes: prefrontal orbitofrontal and ventromedial cortex, dorsolateral prefrontal cortex and anterior cingulate cortex (ACC; for a review, see Martínez-Selva et al., 2006). However, little is known about the differential brain processing of gains and losses that ultimately leads to choosing advantageous decks instead of disadvantages and to succeed in the task. In many gambling tasks, including the IGT, participants generally do not know whether their choice will result in losses or gains until a feedback is provided. These feedback signals can cause specific brain responses that could be detected through the study of electrical brain activity, such as ERPs. It is assumed that brain responses to feedback signals lead to a better adjustment of subsequent behavior, that is, better choices (Holroyd and Coles, 2002). Given the importance of the contingencies associated with each decision for subsequent elections, it would be worthwhile to know the temporal dynamics of the cortical activity related to the results or the feedback of the elections. Feedback signals following gains and losses can cause differential brain processing, which could explain how to guide participants to select mainly advantageous decks. The idea of differential brain processing of gains and losses is strongly supported by experimental data (event-related functional magnetic resonance, fMRI) in gambling tasks such as the IGT (e.g., Lawrence et al., 2009).

Nevertheless, previous research on ERPs elicited during the IGT is scarce and has focused mainly on brain potentials that precede participants’ choices (Bianchin and Angrilli, 2011; Cui et al., 2013). Research on feedback signals has so far focused on the feedback-related negativity (FRN)—a negative wave with a peak latency of about 250–350 ms after feedback onset over frontal and central electrode locations—and the P300 component—a positive wave peaking between 200 ms and 500 ms after feedback onset over parietal and frontal electrode locations—since they are high sensitive to feedback outcome (gain/loss) and to the amount of reward. Moreover, it has been found that FRN amplitudes elicited by losses are larger than those elicited by gains (Bianchin and Angrilli, 2011; Cui et al., 2013). P300 amplitudes seem to increase depending on the result of feedback, with strongest changes elicited by the losses (Cui et al., 2013). Some ERP components are more sensitive to negative than to positive outcomes. In this regard, it has been proposed that the processing of feedback signals as indicated by the FRN and P300 amplitudes could be related to the performance in the IGT. In particular, differences between advantageous and disadvantageous choices in FRN amplitudes would indicate a better discrimination between the two types of decks, and eventually lead to selective choices of the advantageous decks (Cui et al., 2013).

The role of some ERP components, especially short-latency components, is still unclear. Previous research has identified a negative component that reaches a peak between 80 ms and 100 ms at Fz and Cz electrode locations and appears after an error or an unexpected negative outcome mainly in reaction tasks (Arbel and Donchin, 2011). This ERP component has been called error-related negativity (ERN) and seems to mirror the activity of an error detection system (Scheffers and Coles, 2000; Pailing and Segalowitz, 2004; Sailer et al., 2010).

Some authors consider that both ERN and FRN components depend on the same underlying cognitive and neural processes, providing an early processing of feedback outcomes (Nieuwenhuis et al., 2004; Polezzi et al., 2008; Schuermann et al., 2012). Apparently, the FRN component is the feedback variant of the response-locked ERN (Nieuwenhuis et al., 2004; Sailer et al., 2010; Schuermann et al., 2012).

Since the ERN is considered an electrocortical marker caused by incorrect responses during reaction tasks, it is expected that IGT would not be able to elicit it given the time lag between subjects’ responses and feedback signals. Nevertheless, previous studies with gambling and reinforcement learning tasks have reported an early negativity component in response to feedback signals (e.g., Nieuwenhuis et al., 2004; Frank et al., 2005; Eppinger et al., 2008; Schuermann et al., 2012). Given that ERN and FRN reflect the same error-processing system, it is plausible that the first negative deflection elicited by errors or losses in complex decision-making tasks may also reflect an error-processing system such as the ERN in reaction tasks.

A second component of the feedback-related potentials is a positive wave that reaches its maximum at 180–280 ms (P200) over frontal electrode locations. This wave could represent an early processing of several stimulus parameters (predictability, valence, salience) that may be relevant during decision-making processes and subsequent choices (Polezzi et al., 2008; San Martín et al., 2010; Schuermann et al., 2012). In this sense, P200 component has been interpreted as an indicator of early processing of reward feedback signals, with larger amplitudes to losses than to gains (Polezzi et al., 2008; Schuermann et al., 2012). Although some authors consider that P200 is closely related to the P300 component (Falkenstein et al., 2000; Endrass et al., 2012), it seems that P200 could also share some characteristics of the FRN during decision-making tasks.

Regarding the long-latency potentials, it has been suggested that P300 and late ERP components might reflect the allocation of processing resources to motivational or significant stimuli (e.g., Sailer et al., 2010; San Martín et al., 2010) and, therefore, they would be of importance for guiding behavior in subsequent choices. Only few studies have reported changes in very long-latency potentials (latencies longer than 500 ms) on gambling tasks. For instance, Polezzi et al. (2008, 2010) showed that a late negative potential (N500) was higher to unpredictable and negative outcomes. In the same line, Goyer et al. (2008) found that a late wave in the time-window 400–600 ms was more negative to losses than to gains. The authors also suggested that this late component could represent an emotional appraisal of the outcome with influences for the following choices in the task.

Given that decision-making depends on the feedback signals resulting from each single choice and that this feedback causes several ERP components, the goal of this work was to analyze the time course of the cortical activity elicited by gains and losses during the IGT. For this purpose, ERP components in the time-windows 80–350 ms (early negative wave, P200, FRN) and 400–600 ms (P300 and late potentials) were analyzed. To our knowledge, this is the first study covering the five different components that may appear in response to feedback outcomes in the IGT.

According to previous literature, ERPs elicited in the time-window 80–350 ms would reflect an initial analysis of the consequences of the choices, and ERP components with latencies between 400 ms and 600 ms would reflect a more complete analysis of gains and losses, including motivational and emotional processes. The complexity of the IGT requires a great involvement of cortical activity, including the dorsolateral and ventromedial prefrontal cortices and, presumably, a more detailed and slow processing reflected in the ERPs in the time window of 400–600 ms, that might be more related to performance than those elicited in the 80–350 ms time window.

More specifically, we expected that the early negative wave would be sensitive to both gains and losses, whereas losses would provoke larger P200 amplitudes than gains. These components were expected to be followed by an FRN wave with larger amplitudes to losses than to gains. Finally, P300 and late potentials were expected to be differentially influenced by both gains and losses, and also by the amount of money earned or lost, with higher amplitudes to losses than to gains. In addition, we also analyzed the relationship of each of these ERP components with the task performance, since previous research has suggested that some feedback ERPs could be related to IGT performance. We also expected some similarities between the early negative wave and FRN in terms of their amplitude to gains and losses and also in terms of their relationship with task performance.

In addition, we have analyzed brain sources associated with different ERP components. According to previous research, FRN seems to have originated in or near the cingulate cortex and to be the result of a transient decrease in dopaminergic input to the midbrain (Holroyd and Coles, 2002). If the FRN is reflecting the same characteristics in the IGT as in other decision-making tasks, this would indicate a certain similarity in the brain mechanisms involved. The source analyses would provide a more complete picture of the underlying processes in decision-making.

## Materials and Methods

### Participants

Twenty-five female volunteer psychology students, aged between 20 and 32 (mean age 22.4 ± 3.39 years) participated in the present study. Students received course credits for their participation. All participants underwent a psychological interview, including the absence of medical or psychological treatment, psychological disorders or substance abuse. Moreover, the Spanish versions of the Beck Depression Inventory (BDI; mean 4.8 ± 5.2; Beck et al., 1961), the State-Trait Anxiety Inventory (STAI; mean 48.5 ± 6; Spielberger et al., 1970), Sensation Seeking Scale (SSS; mean 20.8 ± 5.9; Zuckerman and Link, 1968) and Barratt Impulsiveness Scale (BIS-11; mean 42.8 ± 9.4; Patton et al., 1995) corroborated that participants fulfilled inclusion criteria. All participants were strongly right-handed as measured by the Edinburgh handedness inventory (EHI; mean 21.2 ± 4.5; Oldfield, 1971), they had no significant neurological history, and they were not receiving psychiatric or pharmacologic treatment. The study was in accordance with the Declaration of Helsinki (World Medical Association, 1991), and written informed consent was obtained from all participants. The protocol was approved by the ethics committee of the University of Balearic Islands.

### Iowa Gambling Task

**Figure 1:**
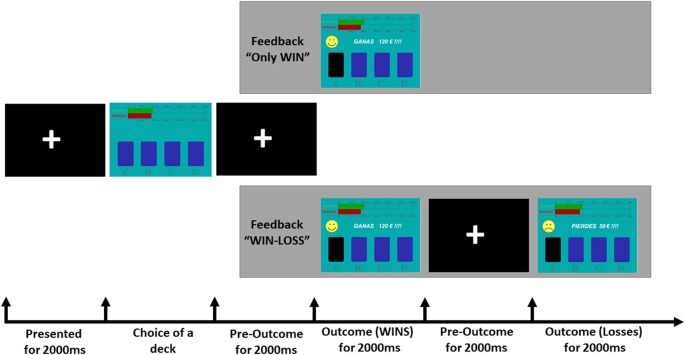
Schematic depiction of the Iowa Gambling Task (IGT) as used in the present study.

We employed a computerized version of the IGT (Bechara et al., 1994) that was modified for ERP recordings (see Figure 1). The IGT was programmed to provide with different amounts of monetary gains after each card choice and to deliver monetary losses of different amounts in specific trials. High amounts of monetary gains and losses were pseudorandomly associated with two decks (disadvantageous), whereas low amounts of monetary gains and losses were associated with the other two decks (advantageous). Thus, the participants could receive four different outcomes: high gain (175€ or 200 €), low gain (25€ or 50€), high loss (−1,000€ or −1,200€) or low loss (−25€ or −50€). The task was designed in such a way that disadvantageous decks also provided high losses. In addition, the frequency of punishments was high (50% of the trials) in two decks (one advantageous and one disadvantageous) and low in the other two decks (10%). The trial started with a fixation cross during 2,000 ms. Then, four decks of cards (A, B, C and D) were displayed and kept on the screen until participants pressed a button corresponding to the selected choice. Next, the fixation cross was again presented during 2,000 ms and participants received a feedback stimulus. Two types of feedback were displayed: “only win” or “win-loss.” In “only win” feedback, a happy yellow face was displayed with the message “WIN” and the positive value of the monetary gain (e.g., +120€; 2,000 ms) followed by a fixation cross (1,000 ms). In “win-loss” feedback, the *gain outcome* (happy yellow face, message “WIN,” monetary gain) was followed by a *loss outcome* (unhappy yellow face with the message “LOSS” and the negative value of the monetary loss, e.g., −50€) for 2,000 ms, and a fixation cross (1,000 ms). For half of participants, A and B decks were designated as advantageous, whereas for the rest of participants they were C and D decks.

Participants completed 100 trials, and they were told that the goal of the task was to obtain monetary gains and to avoid monetary losses (e.g., Bechara et al., 1994).

### Procedure

Data were collected within a single session that lasted 90 min. Participants were verbally informed about the details of the study. A specifically designed, information leaflet was also given to all participants and, after their agreement to participate, a written consent was obtained. The volunteers answered several questionnaires to confirm the inclusion criteria. They were seated comfortably ~1 m in front of a computer screen in a dimly lit, electromagnetically shielded room. Participants were instructed about how to perform the IGT, and 26 practice trials were given before starting the main experiment.

### EEG Recording

Brain electrical activity was recorded with an electrode cap from 60 sites placed according to the international 10–20 system plus two bipolar channels to record electrooculography (EOG) and two electrodes in mastoids as references. Eye movements and blinks were monitored *via* bipolar recordings with electrodes placed above and below the right eye (vertical EOG). Ground was placed anteriorly to the location of the FCz electrode. All impedances were kept below 10 kΩ. The signals were registered by a BrainAmp MR amplifier at a sampling rate of 1,000 Hz, with high and low pass filter settings at 0.10 Hz and 70 Hz, respectively. A 50 Hz notch filter was also applied.

### Data Analyses

#### Behavioral Data

For analysis of task performance, the average over the five blocks of 20 trials were computed for the following two parameters: number of choices from the advantageous and disadvantageous decks, and net scores (computed by subtracting the number of disadvantageous from the number of advantageous choices). The performance was analyzed by an ANOVA with the factor Block (five blocks) as within-subject variable.

The net amount of monetary reward was established by calculating the number of gains and losses obtained by each subject, and analyzed by a 2 × 5 ANOVA, with the within-subject factors *outcome* (gains vs. losses) and *block* (five blocks).

#### EEG Data Pre-processing

EEG recordings were offline processed by using the EEGLAB Toolbox 6.01b (Delorme and Makeig, 2004) running in MATLAB 2008 (Mathworks, Natick, MA, USA). In order to equalize the number of trials in both conditions (“win-loss” outcomes), only those trials with both types of outcomes were selected to be analyzed.

EEG epochs of 900 ms were extracted by using the feedback outcome signals (happy vs. unhappy yellow faces) as trigger onset and 100 ms pre-trigger as baseline. All EEG channels were re-referenced to a common average. Vertical EOG correction was applied by using the Gratton method (Gratton et al., 1983) implemented in EEGLAB (Ocular Correction plugin) with a time window for detection of 20 ms and a criterion for blink detection of 25 μV. In addition, trials with amplitudes greater than ±70 μV were automatically excluded. Finally, trials were visually inspected and excluded if EOG artifacts were still observable. A mean 23.5 of trials of each outcome per participant were accepted to the ERP analyses.

In order to determine those ERP components of interest, individual averages elicited by gains and loss outcomes signals (happy vs. unhappy faces) were separately averaged across all participants (grand averages). From visual inspection at Fz, Cz and Pz, amplitudes in the following latency ranges of interest were examined: 80–180 ms (mean amplitude corresponding to an early negative wave), 180–280 ms (peak amplitude of P200), 280–360 ms (mean amplitude, FRN) and 360–480 ms (peak amplitude of P300), For components later than 480 ms, no clear peak could be identified; therefore, the time-window from 480 ms to 800 ms after outcome onset was selected. Beyond 800 ms no component was selected.

Although the negative wave in the 280–360 ms interval was considered the FRN potential, we took into account previous studies (Toyomaki and Murohashi, 2005; Hajcak et al., 2006; Polezzi et al., 2008; Cui et al., 2013) that have reported that the amplitude of the FRN is affected by P200 (Hajcak et al., 2006; Polezzi et al., 2008). Thus, we considered the difference between the amplitude mean of P200 and amplitude mean of 280–360 ms as the amplitude of the FRN for each ERP and electrode in accordance with the procedure suggested by Hajcak et al. (2006).

Since, we were interested in the “region” effects but not in the individual “electrode” effects, the electrodes were nested within regions. Thus, 33 electrodes were selected to represent six brain regions following the procedure described by Kamarajan et al. (2010): frontal (F3, Fz, and F4), central (FC3, FCz, FC4, C3, Cz, and C4), parietal (P3, Pz, P4, C3P, CPz, and C4P), occipital (O1, Oz, O2, Po3, Poz, and Po4), left temporal (FT7, T7, TP7, CP5, P7, and P5) and right temporal (FT8, T8, TP8, CP6, P8, and P6). The effects of outcome signals (gains vs. losses) on the amplitudes of the early negative wave, P200, FRN, P300 and late potential components were tested by using ANOVAs for repeated measures at each brain region.

#### Relation Between ERP Amplitudes and Task Performance

To analyze the relation between ERP amplitudes and task performance (net scores, number of advantageous and disadvantageous choices, amount of money obtained), bivariate Pearson correlations were computed for Fz, Cz, and Pz.

All statistical analyses were performed by using the Statistical Package for Social Sciences (SPSS) 15 software. Greenhouse-Geisser corrections were applied when necessary, and *post hoc* pairwise comparisons were performed using the Bonferroni correction with a significant level of *p* < 0.05. The reported significance results are presented with the original degrees of freedom and a measure of the effect size.

#### Brain Source Analyses

Brain source localization of the ERP components elicited by gains and losses was computed by using the BrainStorm 3.1 software (Tadel et al., 2011). Artifact-free ERP data from 60 electrodes were used to obtain source localization maps corresponding to the two types of outcome (gains/losses). The inverse problem solution was computed by using the Standardized Low Resolution Electromagnetic Tomography (sLORETA) software. The head was modeled using the MNI-Colin25 high-resolution T1-weighted MR images, and a 3-shell sphere Berg approximation representing the brain. The cortical surface was parsed, represented as a high-density mesh of vertices, and subsequently down-sampled to 1,516 vertices and electrode positions were approximated based on a template electrode position file. Current source density estimates were *z*-score normalized relative to the baseline (−100 ms to 0 ms prior to outcome onset). Each source map was thresholded at *p* < 0.05 value relative to the post-outcome distribution of all vertices in each time interval, and a cluster threshold (10 vertices connected) was applied.

## Results

### IGT Performance

**Figure 2:**
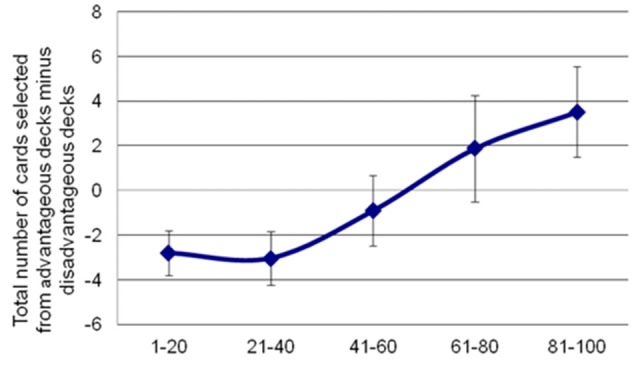
Participants’ performance in the IGT along the 100 trials. Net scores were calculated by subtracting the number of advantageous choices from the number of disadvantageous ones. Error bars represent the standard error of the mean.

Analyses of behavioral parameters revealed that subjects improved their performance (i.e., they selected more advantageous than disadvantageous decks) as the task progressed (see Figure 2). The statistical analyses yielded a significant main effect of Block (*F*_(4,96)_ = 6.44, *p* = 0.003, $\eta_{\text{p}}^{2}$ = 0.21). Bonferroni *post hoc* tests showed significant differences in task performance between block 5 and block 1 (*p* = 0.034), block 2 (*p* = 0.004) and block 3 (*p* = 0.009).

These results were confirmed when the amount of monetary reward during gain and loss trials were compared along the five blocks. The 2 × 5 repeated-measures ANOVA revealed significant differences due to outcome (*F*_(1,23)_ = 417.75, *p* < 0.001, $\eta_{\text{p}}^{2}$ = 0.94). Overall, participants obtained more gains than losses during the task. No significant differences due to blocks or to the interaction were found (all *p*s > 0.05).

### Time Course of ERPs to Gains and Losses

**Figure 3:**
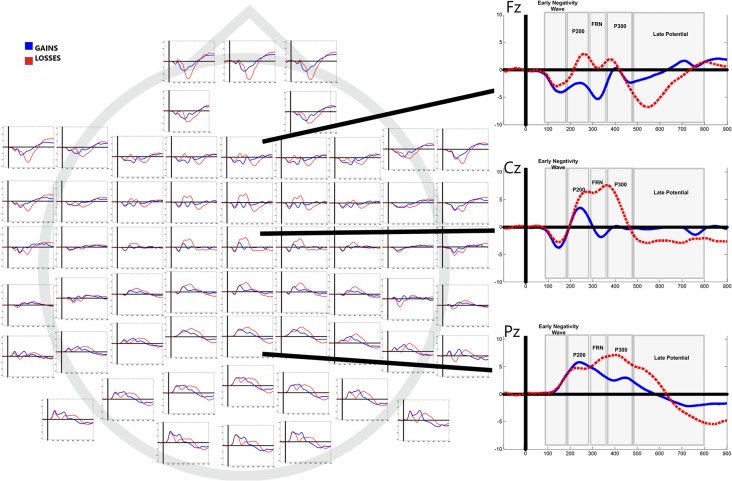
Grand averaged event-related potentials (ERPs) to feedback of gains and losses in all electrode sites. Right column shows average responses in Fz, Cz and Pz.

Figure 3 shows grand average waveforms separated by outcome (gains vs. losses). Mean values of mean amplitudes and electrode localization are displayed in Table 1.

**Table 1:** Means and standard deviations (in parentheses) of area under curve of the event-related potentials (ERPs) according to interval and cerebral regions.

	Localization						
Interval (ms)	Valence	Frontal	Central	Parietal	Occipital	Left temporal	Right temporal
Early negativity wave	Win	−4.98 (0.32)	−4.54 (0.29)	−1.45 (0.14)	−0.25 (0.26)	−1.84 (0.17)	−2.08 (0.14)
80–180	Loss	−5.31 (0.40)	−4.77 (0.31)	−2.11 (0.21)	−0.96 (0.43)	−3.25 (0.29)	−4.01 (0.30)
P200	Win	−0.82 (0.27)	1.62 (0.21)	4.80 (0.23)	7.92 (0.32)	2.03 (0.21)	1.91 (0.20)
180–280	Loss	3.17 (0.42)	4.41 (0.42)	4.92 (0.33)	8.06 (0.58)	1.92 (0.35)	0.55 (0.35)
FRN	Win	−5.78 (0.39)	−3.04 (0.59)	1.46 (0.21)	2.90 (0.33)	−0.83 (0.17)	−0.56 (0.16)
280–360	Loss	−4.54 (0.66)	0.10 (0.58)	1.49 (0.35)	0.004 (0.51)	−3.55 (0.46)	−4.76 (0.43)
P300	Win	1.19 (0.26)	1.53 (0.15)	3.39 (0.19)	4.43 (0.34)	2.77 (0.18)	2.74 (0.20)
360–480	Loss	1.68 (0.44)	5.51 (0.61)	7.55 (0.55)	6.83 (0.61)	3.59 (0.34)	3.98 (0.30)
Late potential	Win	0.26 (0.17)	−0.02 (0.11)	−0.56 (0.14)	−2.53 (0.24)	0.76 (0.13)	0.21 (0.10)
480–800	Loss	−1.69 (0.44)	−1.70 (0.24)	−0.02 (0.27)	−0.48 (0.42)	1.47 (0.22)	0.50 (0.27)

#### Early Negative Wave (80–180 ms Interval)

ANOVAs revealed that amplitudes of the early negative wave were smaller for gains than for losses at parietal (*F*_(1,24)_ = 7.83, *p* = 0.02 $\eta_{\text{p}}^{2}$ = 0.24), left temporal (*F*_(1,24)_ = 18.87, *p* < 0.001 $\eta_{\text{p}}^{2}$ = 0.44) and right temporal brain regions (*F*_(1,24)_ = 33.69, *p* < 0.001 partial $\eta_{\text{p}}^{2}$ = 0.58; see Table 1).

#### P200 (180–280 ms Interval)

ANOVAs revealed that P200 amplitudes were smaller for gains than for losses at frontal (*F*_(1,24)_ = 69.91, *p* < 0.001 $\eta_{\text{p}}^{2}$ = 0.74) and central brain regions (*F*_(1,24)_ = 42.83, *p* < 0.001 $\eta_{\text{p}}^{2}$ = 0.64; see Table 1).

#### FRN (280–360 ms Interval)

ANOVAs revealed that FRN amplitudes were greater for gains than for losses at central (*F*_(1,24)_ = 63.88, *p* < 0.001 $\eta_{\text{p}}^{2}$ = 0.72), occipital (*F*_(1,24)_ = 24.01, *p* < 0.001 $\eta_{\text{p}}^{2}$ = 0.50), left temporal (*F*_(1,24)_ = 53.29, *p* < 0.001 $\eta_{\text{p}}^{2}$ = 0.68) and right temporal (*F*_(1,24)_ = 25.25, *p* < 0.001 $\eta_{\text{p}}^{2}$ = 0.51).

**Figure 4:**
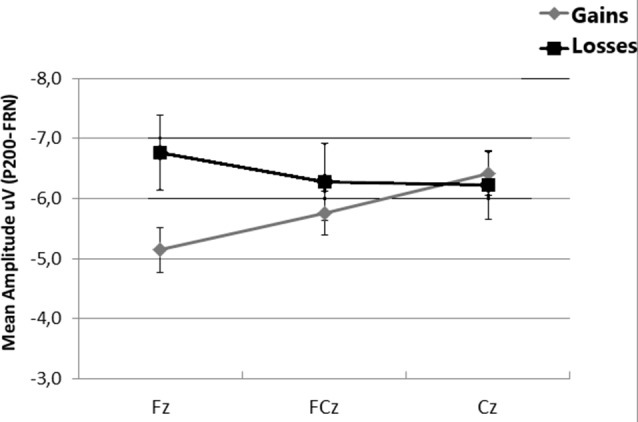
Mean amplitudes of feedback related negativity (FRN) at Fz, FCz and Cz, calculated as the difference between the mean amplitude the 180–280 ms interval and the mean amplitude in the 280–360 ms interval. Error bars represent the standard error of the mean.

When FRN was computed as the difference between P200 amplitudes and the mean amplitude between 280 ms and 360 ms after onset, a *t*-test indicated that differences between gains and losses were largest at Fz (*t* = 2.60, *df* = 24, *p* = 0.016, one-tailed). Non-significant differences were found at FCz and Cz (all *p*s > 0.05; see Figure 4).

#### P300 (360–480 ms Interval)

ANOVAs on P300 revealed that losses elicited enhanced P300 amplitudes than gains at central (*F*_(1,24)_ = 44.42, *p* < 0.001 $\eta_{\text{p}}^{2}$ = 0.64), parietal (*F*_(1,24)_ = 48.58, *p* < 0.001 $\eta_{\text{p}}^{2}$ = 0.66), occipital (*F*_(1,24)_ = 11.84, *p* < 0.001 $\eta_{\text{p}}^{2}$ = 0.33), and right temporal (*F*_(1,24)_ = 9.07, *p* < 0.001, $\eta_{\text{p}}^{2}$ = 0.27; see Table 1).

#### Late Potential (450–800 ms Interval)

ANOVAs revealed that losses elicited more positive amplitudes in this latency range than gains at frontal (*F*_(1,24)_ = 14.55, *p* < 0.001 $\eta_{\text{p}}^{2}$ = 0.37), central (*F*_(1, 24)_ = 31.80, *p* < 0.001 $\eta_{\text{p}}^{2}$ = 0.57), occipital (*F*_(1,24)_ = 13.58, *p* < 0.001, $\eta_{\text{p}}^{2}$ = 0.36), and left temporal (*F*_(1,24)_ = 6.39, *p* = 0.018 $\eta_{\text{p}}^{2}$ = 0.21; see Table 1).

### Correlations Between ERP Amplitudes and IGT Performance

Significant positive correlations were found between the number of advantageous selections in the IGT and amplitudes of early negative wave elicited by gains at Fz (*r* = 0.43, *p* = 0.029), and Cz (*r* = 0.50, *p* = 0.011). The early negative amplitudes elicited by losses at Cz also correlated positively with the number of advantageous selections (*r* = 0.45, *p* = 0.024).

Significant positive correlations were found between the number of disadvantageous selections and P200 amplitudes elicited by losses at Fz (*r* = 0.52, *p* = 0.007) and Cz (*r* = 0.44, *p* = 0.024). FRN amplitudes elicited by gains also showed a positive correlation with the number of advantageous selections at Pz (*r* = 0.56, *p* = 0.004). Finally, the mean amplitude of the late potential elicited by gains at Pz were positively correlated with the number of advantageous selections (*r* = 0.41, *p* < 0.038).

No significant correlations were found between net scores and amount of monetary reward.

### Source Localization

**Figure 5:**
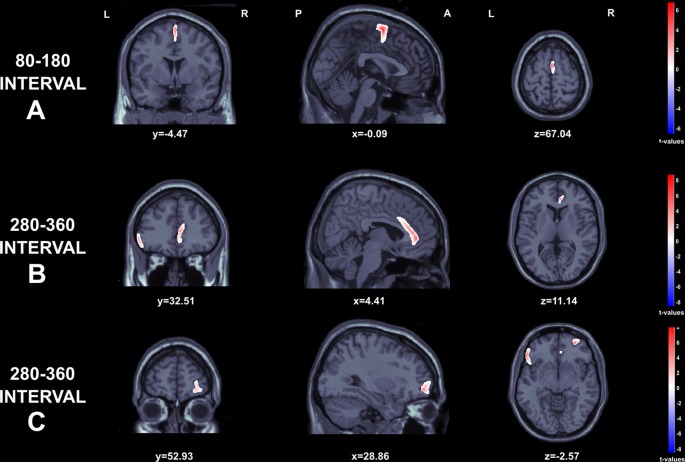
Graphical representation of the Standardized Low Resolution Electromagnetic Tomography (sLORETA) *t*-statistics comparing the ERPs for gains and losses at the time point of the individual peak over 80–180 s interval **(A)** and 280–360 s intervals (**B** and **C**). Red color indicates local maxima of increased electrical activity for loss compared to winresponses in an axial, a sagittal and a coronal slice through the reference brain.

SLORETA *t*-test maps for comparisons among gains and losses are depicted in Figure 5. The data from two participants were excluded due to electrode problems. Regarding the early negative wave, greater source activity was observed to losses in comparison to gains over the Supplementary motor area (*t*_(44)_ = 6.67, *p* < 0.001; MNI: 1.94–8.65 69.87) extending to middle cingulum (MNI: 1.67 −7.89 48.84; Figure 5A). For the FRN component, we found significantly higher activation in the ACC (*t*_(44)_ = 1.01, *p* < 0.05 ; MNI: 6.13 35.50 11.22), inferior frontal gyrus, corresponding to Brodmann area 47 (BA 47; *t*_(44)_ = 3.22, *p* < 0.05; MNI: −56.42 26.07 −3.40), and right middle orbito-frontal gyrus (*t*_(44)_ = 3.87, *p* < 0.001 MNI: 29.84 50.97 −3.68; see Figures 5B,C).

## Discussion

ERPs elicited by gains and losses during the IGT were analyzed in the time window 80–800 ms, as well as the relationship between the amplitudes of several ERP components and task performance. Given that previous literature has found most relevant results at medial fronto-central (early negative wave, P200, FRN) and parietal (P300) electrode locations, this discussion will deal mainly with results obtained at Fz, Cz and Pz electrodes (e.g., Hajcak et al., 2005; Hewig et al., 2011).

Our data indicated that the processing of outcome signals during the IGT started with an early negative wave, indicating that the evaluation of the consequences of a given choice starts very early after the feedback stimulus. By contrast, the processing of losses (negative outcomes) started around 200 ms with larger P200 amplitudes elicited by losses than by gains. The FRN showed the well-known effect of increased magnitude to losses than to gains, followed by larger P300 amplitudes elicited by losses than by gains. These results can be interpreted as reflecting greater motivational significance of losses in comparison to gains. In addition, losses elicited greater processing resources, as reflected by the larger amplitude of the late ERP components beyond P300.

Our prediction that ERPs in the 400–600 ms time window would be more related to performance than those ERPs with shorter latencies was not fulfilled. In fact, all ERPs, with the exception of P300, were related to task performance in terms of the numbers of gains and losses. In the following sections, results from the five ERPs will be closely discussed.

### Early Negative Wave

An early negative wave was found after both gains and losses, corresponding to an early negativity in the visual N1 range (Luck, 2005). Negative outcomes in the IGT (losses) elicited more negativity than positive outcomes (gains) at parietal, left and right temporal electrodes. By contrast, no significant differences were observed at frontal and central electrode locations. Amplitude and correlation analyses also revealed that this early brain response may reflect a general evaluative process rather than a specific processing of negative feedback outcomes. In agreement with previous data, brain sources for this ERP response were found at the supplementary motor area, reaching the medial cingulate cortex (Roger et al., 2010).

The amplitudes of this early negative wave at FCz and Cz were correlated with the number of gains and losses, as well as the number of advantageous choices. This was partially in agreement with previous results by Frank et al. (2005), showing a significant relationship between the amplitude of an early negative wave and good decisions during a reinforcement learning task. Thus, it seems that this short-latency wave appears after behaviorally relevant stimuli related to the task. This is compatible with the idea of an initial assessment of the consequences of the choice without distinguishing between positive (gains) and negative feedback outcomes (losses).

### P200

The first positive wave (P200) elicited larger amplitudes to losses than to gains at frontal and central electrode locations. This was in accordance with previous studies in decision-making tasks (Polezzi et al., 2008; Schuermann et al., 2012). The P200 amplitude to losses at FCz and Cz was positively correlated with the number of losses, thus reinforcing the idea of P200 as an early component mainly related to the processing of negative feedback signals, error awareness and error-related information (Steinhauser and Yeung, 2010, 2012). Polezzi et al. (2008) found that this component was directly related to the predictability of the outcomes, with higher amplitudes to unpredictable outcomes. In this line, the uncertainty of the IGT results in higher P200 amplitudes to losses. In contrast with the early negative wave, P200 represents an early differentiation between gain and loss feedback, and its amplitude was related with the selection of the disadvantageous decks.

### FRN

A negative deflection, similar to the FRN, in the time window 280–360 ms that interrupted an ongoing positive wave was observed in frontal, central, occipital and right and left temporal electrodes to both types of outcome signals (gains and losses). Negativity was higher in loss trials at occipital and both left and right temporal electrodes. However, and contrary to our predictions, loss signals elicited more positive amplitudes than gains at frontal and central electrodes. When differential measures between the previous P200 and the FRN were taken into account, losses resulted in a more negative wave than gains at frontal electrodes but not at central electrodes. This was in agreement with Cui et al. (2013) who found larger effects on FRN between gains and losses at Fz, and in partial agreement with Bianchin and Angrilli (2011) who reported larger FRN components to loss than to gains at both Fz and FCz during the performance of the IGT.

Significant positive correlations were found between FRN amplitudes to gains at Pz and the number of advantageous choices, indicating that the larger the amplitude of the ERP elicited by gains, the better was the performance in the IGT. Interestingly, source analysis revealed higher activation in the ACC, inferior frontal gyrus (BA 47) and right middle orbito-frontal gyrus. The ACC is the region where the FRN is supposed to be generated, thus confirming previous research (Gehring and Willoughby, 2002). These data are in accordance with functional imaging studies during performance of the IGT that point to a cluster of brain regions involved in the processing of the consequences of the choices: ACC, the ventromedial prefrontal cortex and its orbitofrontal section, and the inferior frontal gyrus (Fukui et al., 2005; Northoff et al., 2006; Li et al., 2010; Ma et al., 2015; Wang et al., 2017).

### P300

Losses elicited larger P300 amplitudes than gains at all electrodes. This is in accordance with previous reports that have found higher amplitudes in this component to losses than to gains (Frank et al., 2005; Cohen et al., 2007; San Martín et al., 2010; Schuermann et al., 2012). In addition, our results were in agreement with data from Cui et al. (2013) who found in the IGT larger P300 amplitudes to losses than to gains. Nevertheless, other authors found larger P300 amplitudes to gains than to losses in gambling tasks other than the IGT (e.g., Hajcak et al., 2005). Since P300 amplitude is related to the motivational significance of the result of the choice, this positive wave could reflect a late evaluation process more sensitive to losses than to gains in the IGT.

### Late Potential

Lastly, in accordance with some researchers (Polezzi et al., 2008, 2010) a late negative component in the 450–800 ms time window, similar to N500, appeared as a response to losses at frontal and central regions, while less negativity dominated in the reaction to gains. More negativity to losses than to gains has also been reported by Goyer et al. (2008). In addition, the amplitude of this late component to gains at Pz positively correlated with the number of gains.

Interestingly, the amplitude of the three negative waves (early negative wave, FRN and the long-latency wave) correlated with the number of gains, and two of them, early negativity and FRN, had the same source in or near the ACC, suggesting a similar origin and function, as well as the involvement of the medial prefrontal cortex, especially the anterior cingulate, in decision making.

### Similarities Between P200 and P300

P200 and P300 were more sensitive to losses and behaved in a parallel way. This result gives support to the hypothesis suggesting that P200 shares some features with the classic stimulus-locked P300, and that these two feedback-related positive waves reflect the same processes related to the conscious recognition or the motivational significance of the error (Falkenstein et al., 2000; Steinhauser and Yeung, 2010; Arbel and Donchin, 2011; Endrass et al., 2012). P200 seems to be mainly indicative of an early reaction to losses or worse than expected outcomes and associated to increased attention and greater arousal levels, while P300 would be indicative of additional information processing and of the motivational significance of the loss (San Martín et al., 2010; Schuermann et al., 2012). In the IGT, P200 would appear as an early component reflecting an initial processing of mainly negative feedback signals, while P300 would reflect a conscious processing of either the motivational significance of losses or of the relative frequency of the different outcomes.

## Conclusion

Our study extends previous results on the ERPs evoked by feedback signals in decision-making tasks to the whole range of electrocortical activity. The processing of loss feedback seems to be an important feature in the performance of the IGT and, consequently, losses rather than gains seem to guide the selection of the decks, an aspect that should be addressed in further studies.

This characterization of the ERP components associated to feedback may be helpful in order to discriminate the processing steps of the feedback received after an option is chosen, and that might be necessary to guide the behavior in subsequent choices. Further research is needed in order to test whether a failure in some of these processing steps, as revealed by ERPs, may result in a deficit in decision making, as may be happening in individuals with several pathological conditions (e.g., drug addiction, etc.). A limitation that should be addressed in further works is that only female participants were studied. Gender differences in the performance of the IGT have been reported by several authors, with the consistent finding that men generally tend to choose the advantageous decks more frequently and outperform women (Byrne and Worthy, 2016), and this calls for the need to include male participants in further studies.

## Ethics Statement

The study was in accordance with the Declaration of Helsinki (World Medical Association, 1991), with written informed consent from all subjects. The protocol was approved by the ethics committee of University of Balearic Islands.

## Author Contributions

All authors provided a substantial contribution to the design and interpretation of the protocol and guidance, as well as writing sections of drafts, revising based on comments received, and approving the final version. PM, CW and MM conducted the analysis, drafted and revised the protocol. JM-S and JS-N conducted the drafts and corrections of this article. All authors read and approved the final manuscript.

## Conflict of Interest Statement

The authors declare that the research was conducted in the absence of any commercial or financial relationships that could be construed as a potential conflict of interest.
